# Sustained inhibition of NPY/AgRP neuronal activity by FGF1

**DOI:** 10.1172/jci.insight.160891

**Published:** 2022-09-08

**Authors:** Eunsang Hwang, Jarrad M. Scarlett, Arian F. Baquero, Camdin M. Bennett, Yanbin Dong, Dominic Chau, Jenny M. Brown, Aaron J. Mercer, Thomas H. Meek, Kevin L. Grove, Bao Anh N. Phan, Gregory J. Morton, Kevin W. Williams, Michael W. Schwartz

**Affiliations:** 1 Department of Internal Medicine, Center for Hypothalamic Research, University of Texas Southwestern Medical Center at Dallas, Dallas, Texas, USA.; 2 Department of Medicine, University of Washington Medicine Diabetes Institute, Seattle, Washington, USA.; 3 Department of Pediatric Gastroenterology and Hepatology, Seattle Children’s Hospital, Seattle, Washington, USA.; 4 Obesity Research, Novo Nordisk Research Center Seattle, Seattle, Washington, USA.; 5 University of Copenhagen, Novo Nordisk Foundation Center for Basic Metabolic Research, Copenhagen, Denmark.; 6 Discovery Technologies & Genomics, Novo Nordisk Research Centre Oxford, Oxford, United Kingdom.

**Keywords:** Endocrinology, Neuroscience, Diabetes, NPY, Obesity

## Abstract

In rodent models of type 2 diabetes (T2D), central administration of FGF1 normalizes elevated blood glucose levels in a manner that is sustained for weeks or months. Increased activity of NPY/AgRP neurons in the hypothalamic arcuate nucleus (ARC) is implicated in the pathogenesis of hyperglycemia in these animals, and the ARC is a key brain area for the antidiabetic action of FGF1. We therefore sought to determine whether FGF1 inhibits NPY/AgRP neurons and, if so, whether this inhibitory effect is sufficiently durable to offer a feasible explanation for sustained diabetes remission induced by central administration of FGF1. Here, we show that FGF1 inhibited ARC NPY/AgRP neuron activity, both after intracerebroventricular injection in vivo and when applied ex vivo in a slice preparation; we also showed that the underlying mechanism involved increased input from presynaptic GABAergic neurons. Following central administration, the inhibitory effect of FGF1 on NPY/AgRP neurons was also highly durable, lasting for at least 2 weeks. To our knowledge, no precedent for such a prolonged inhibitory effect exists. Future studies are warranted to determine whether NPY/AgRP neuron inhibition contributes to the sustained antidiabetic action elicited by intracerebroventricular FGF1 injection in rodent models of T2D.

## Introduction

In addition to its role as a tissue growth factor involved in functions ranging from brain development to angiogenesis, FGF1 exerts a highly durable glucose-lowering action following central administration in rodent models of type 2 diabetes (T2D) (1–3). In particular, a single intracerebroventricular (i.c.v.) injection of FGF1 induces remission of diabetic hyperglycemia that lasts for weeks or even months in both mouse (*Lep^ob/ob^* and *LepR^db/db^*) and rat (Zucker diabetic fatty [ZDF]) models of T2D. The mediobasal hypothalamus (MBH) is a key brain area involved in glucose homeostasis that is highly responsive to FGF1 following i.c.v. injection (4). Moreover, the effect of i.c.v. FGF1 injection to elicit sustained remission of diabetic hyperglycemia can be recapitulated by microinjection of a much lower FGF1 dose directly into the MBH (4), implicating this brain area in the mechanism underlying sustained glucose lowering following i.c.v. FGF1 administration.

The arcuate nucleus (ARC) is situated adjacent to the floor of the third ventricle in the MBH. The ARC contains 2 neuron subsets with opposing effects on energy balance that interact to determine the activity of the hypothalamic melanocortin system. On the one hand are neurons that coexpress agouti-related peptide (AgRP) and neuropeptide Y (NPY) (referred to as NPY/AgRP neurons hereafter) that when activated, reduce signaling via the melanocortin 4 receptor (Mc4r; AgRP is an inverse agonist of this receptor). On the other hand, opposing this action are adjacent neurons that express proopiomelanocortin (POMC) and release α-melanocyte–stimulating hormone (α-MSH), a ligand that binds to and activates Mc4r (5, 6). Under usual circumstances, the activity of these 2 neuronal subsets is reciprocally regulated, such that when NPY/AgRP neurons are activated, POMC neurons are inhibited and vice versa. In states of negative energy balance (e.g., fasting), for example, NPY/AgRP neuron activation combines with POMC neuron inhibition to potently reduce Mc4r signaling and, thereby, promote a state of positive energy balance, leading to recovery of lost weight.

Glucose metabolism is also impaired by NPY/AgRP neuron activation (7–12), while conversely, blood glucose, food intake, and body weight are each reduced when NPY/AgRP neurons are silenced or ablated, including in mouse models of T2D (13–15). Because elevated hypothalamic *Agrp* and *Npy* mRNA levels are a characteristic finding in rodent models of diabetes (16), we wondered whether NPY/AgRP neuron inhibition might be a target for the antidiabetic action of FGF1 in the MBH. Support for this hypothesis includes evidence from 3 different mouse models of T2D, which show that sustained glucose lowering induced by i.c.v. FGF1 injection is blocked when Mc4r signaling is disrupted by either genetic or pharmacological means (16). These findings suggest that for FGF1 to exert its central antidiabetic action, melanocortin signaling must increase, and this can only happen by inhibiting NPY/AgRP neurons, activating POMC neurons, or some combination of the two (5, 6).

Consistent with this notion is recent evidence from the Kievit lab, which shows that, in an ex vivo brain slice preparation, POMC neurons are depolarized following bath application of FGF1 (1, 2, 17); additional findings implicate NPY/AgRP neurons as targets for the action of FGF1 as well. Specifically, a study in diabetic *Lep^ob/ob^* mice demonstrated that at the transcriptional level NPY/AgRP neurons are the most FGF1-responsive hypothalamic neuronal cell type (16); this study also showed that expression of both *Npy* and *Agrp* mRNA is reduced for weeks following a single i.c.v. FGF1 injection, suggestive of a sustained inhibitory response (16). Another line of evidence involves perineuronal nets (PNNs), extracellular matrix specializations that enmesh and thereby regulate the function of distinct neuronal subsets across many brain areas. We reported recently that most NPY/AgRP neurons are enmeshed by PNNs (18) and, subsequently, that these PNNs are depleted in the in ZDF rat model of T2D (19). Moreover, these ARC PNNs are reassembled following i.c.v. FGF1 injection, and this restorative effect of FGF1 appears to be required for sustained diabetes remission to be induced in these animals (19).

Multiple convergent findings therefore support a model whereby FGF1 action in the MBH increases melanocortin signaling, at least in part, by inhibiting NPY/AgRP neurons. In this work, we combined histochemical and electrophysiological tools to investigate effects of FGF1 on NPY/AgRP neuron function both in WT mice and in NPY/AgRP-reporter mice that were crossed onto the *Lep^ob/ob^* mouse background, enabling us to identify and target NPY/AgRP neurons for electrophysiological recordings in a highly FGF1-responsive mouse model of T2D. To assess the onset and duration of the response of these neurons to FGF1, studies were carried out both acutely and 2 weeks after i.c.v. injection.

## Results

### Acute FGF1 administration inhibits NPY/AgRP neurons.

**Figure 1:**
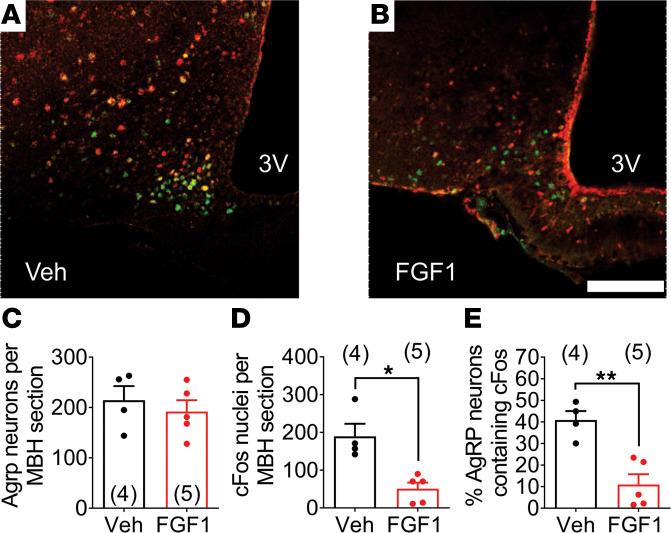
FGF1 inhibits fasting-induced activation of NPY/AgRP neurons. Immunohistochemical detection of *Agrp^Cre:GFP^* (green), cFos (red), and colocalization of GFP and cFos in the ARC of *Agrp^Cre:GFP^* mice fasted for 24 hours 90 minutes after i.c.v. injection of vehicle or FGF1 (3 μg). Representative coronal images from (**A**) vehicle-treated and (**B**) FGF1-treated mice. Quantitation of (**C**) the total number of NPY/Agrp neurons, (**D**) the total number of cFos^+^ cells, and (**E**) the percentage of NPY/AgRP neurons that coexpress cFos. Scale bar: 100 μm. *n* = 4–5 group; mean ± SEM. *P < 0.05, **P < 0.01, unpaired *t* test. 3V, third ventricle.

As a first step to investigate whether NPY/AgRP neurons are inhibited by the action of FGF1, we utilized transgenic *Agrp^Cre:GFP^* mice to enable detection of cFos protein, a marker of neuron activation, in labeled NPY/AgRP neurons. This study was performed in nondiabetic mice that were fasted for 24 hours to increase baseline expression of cFos in NPY/AgRP neurons. Mice were euthanized 90 minutes after i.c.v. injection of either vehicle or FGF1 (3 μg), with representative images from treated animals presented in Figure 1, A and B. Although the total number of AgRP^+^ neurons was comparable across all mice tested (Figure 1C), we found that the total number of both cFos^+^ cells and cFos^+^AgRP^+^ neurons across the entire rostral-to-caudal extent of the ARC was markedly reduced in fasted mice receiving i.c.v. injection of FGF1 compared with saline-injected controls (Figure 1, D and E). These findings suggest that in fasted normal mice AgRP neurons are rapidly inhibited following i.c.v. FGF1 administration, in agreement with our prior observation that i.c.v. FGF1 decreases hypothalamic *Agrp* and *Npy* mRNA levels (16).

**Figure 2:**
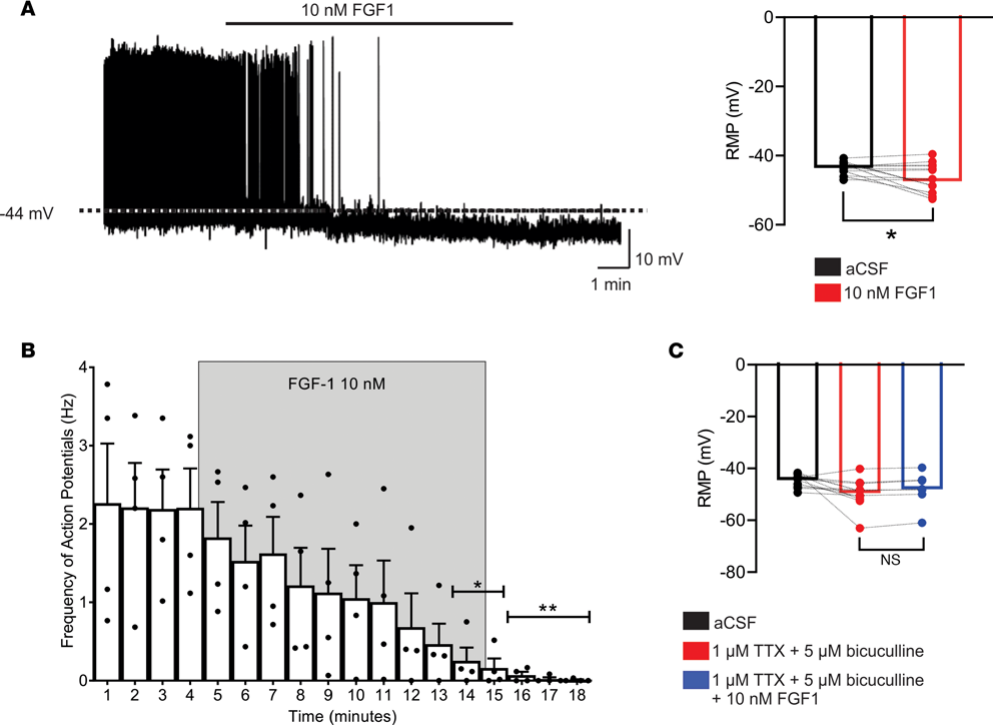
FGF1 inhibits NPY/AgRP neurons by an indirect mechanism. (**A**) Representative trace of an NPY/AgRP neuron, illustrating a membrane hyperpolarization in response to FGF1 (10 nM). The bar graph shows the magnitude of FGF1 responses in NPY/AgRP neurons. (**B**) FGF1 (10 nM) decreased the action potential frequency in NPY/AgRP neurons progressively over a 10-minute period. (**C**) FGF1 failed to change the membrane potential of NPY/AgRP neurons in the presence of 1 μM tetrodotoxin (TTX) and 5 μM bicuculline. Data are shown as mean ± SEM. *P < 0.05, **P < 0.01, (**A**) paired *t* test; (**B**) ANOVA and Dunnett’s multiple comparison; and (**C**) ANOVA and Bonferroni’s correction as post hoc. The dashed lines indicate the resting membrane potential (RMP). Seven mice were used to generate data for **A** (*n* = 13), **B** (*n* = 4), and **C** (*n* = 7).

We then examined the response of NPY/AgRP neurons to bath application of FGF1 ex vivo using whole-cell patch-clamp electrophysiology in nondiabetic C57BL/6 mice (in which the neurons are marked by GFP expressed under the control of the *Npy* promoter; ref. 20). When K^+^ was used as the major cation in the recording pipette, the resting membrane potential (RMP) of ARC NPY/AgRP neurons was –43.7 ± 0.5 mV. Bath application of FGF1 (10 nM in artificial CSF [aCSF]) hyperpolarized the membrane potential in 5 of 13 NPY neurons (38.5% of NPY neurons targeted, *P =* 0.0009; Figure 2A) in a dose-responsive manner (Supplemental Figure 1; supplemental material available online with this article; https://doi.org/10.1172/JCI160891DS1). In the subset of these neurons that were also active (baseline firing rate, ≥0.5 Hz), we observed a progressive decrease in the frequency of action potentials over time (*n =* 4, *P =* 0.0205 at 14 minutes; *P =* 0.0135 at 15 minutes; *P =* 0.0088 at 16 minutes; *P =* 0.0069 at 17 minutes; *P =* 0.0065 at 18 minutes; 1-way ANOVA, Figure 2B). Cumulatively, the overall membrane potential from all NPY/AgRP neurons, including responsive and nonresponsive cells, was significantly hyperpolarized in response to FGF1 bath application (*n =* 13; *P =* 0.038 paired *t* test; Figure 2A). Interestingly, antagonism of GABAergic neurotransmission prevented the hyperpolarization of NPY/AgRP neurons (Figure 2C), in agreement with our finding of increased GABAergic synaptic transmission during acute FGF1 administration (Supplemental Figure 2). Together, these findings suggest that FGF1 inhibits NPY/AgRP neurons indirectly via a mechanism requiring increased GABAergic input.

### Sustained inhibition of NPY/AgRP neurons following i.c.v. FGF1 administration to NPY^hrGFP^:Lep^ob/ob^ mice.

**Figure 3:**
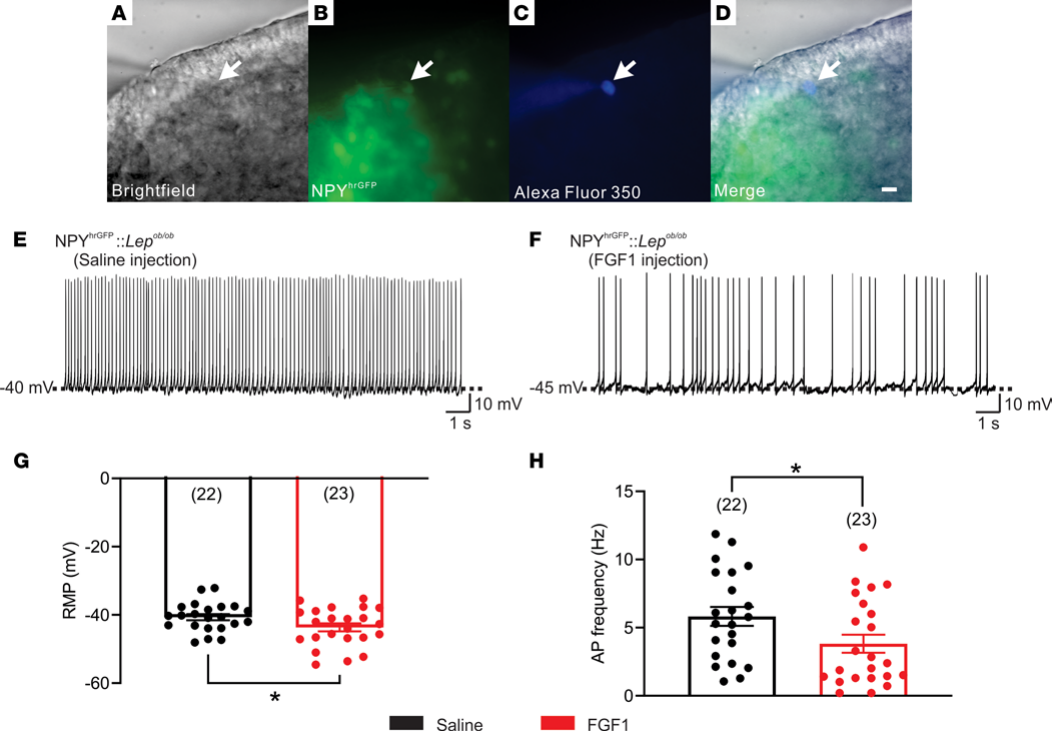
FGF1 persistently inhibits NPY/AgRP neurons in *Lep^ob/ob^* mice. (**A**) Bright-field illumination of an NPY neuron from *Lep^ob/ob^* mice. (**B**) The same neuron under FITC (hrGFP) and (**C**) Alexa Fluor 350 illumination. (**D**) Merged image of targeted NPY neuron. Arrows indicate the targeted cell. Scale bar: 50 μm. (**E** and **F**) Current-clamp recording of an NPY neuron, showing the resting membrane potential from male *Lep^ob/ob^* mice receiving (**E**) saline or (**F**) FGF1. (**G** and **H**) Histograms demonstrate (**G**) the average resting membrane potential and (**H**) action potential frequency of NPY neurons from male *Lep^ob/ob^* mice injected with saline (black, *n* = 22, from 3 mice) or FGF1 (red, *n* = 23, from 3 mice). Data were taken from NPY neurons of male NPYhrGFP:*Lep^ob/ob^* mice and are expressed as mean ± SEM. * *P <* 0.05, unpaired *t* test compared with the saline group. The number of neurons studied for each group is in parentheses.

Next, we determined whether sustained diabetes remission induced by i.c.v. FGF1 administration is associated with a persistent decrease in the activity of arcuate NPY/AgRP neurons. In this experiment, we used whole-cell patch-clamp electrophysiological recordings on NPY neurons from male NPY^hrGFP^:*Lep^ob/ob^* and *Lep^ob/+^* mice (Figure 3, A–D). Based on previous reports describing long-lasting hypothalamic effects following i.c.v. FGF1 administration in mice (1, 2, 17), these studies were performed 2 weeks after a single i.c.v. injection of either saline or FGF1 (3 μg). As expected based on our previous observations (2, 3), *Lep*^ob/ob^ mice receiving FGF1 (3 μg, i.c.v.) exhibited transient reductions of food intake and body weight that returned to baseline within days after injection, whereas no such effects were observed in *Lep^ob/ob^* mice receiving i.c.v. saline (Supplemental Figure 3). Consistent with earlier findings (21), NPY/AgRP neurons from obese diabetic *Lep^ob/ob^* mice receiving i.c.v. saline trended toward an increased AP frequency (Figure 3, E, F, and H) compared with nonobese, nondiabetic *Lep^ob/+^* mice receiving i.c.v. saline (*P =* 0.07, Supplemental Figure 4H), despite the absence of a similar trend in RMP between groups (*P =* 0.18, Supplemental Figure 4G). Moreover, we observed that, 2 weeks following a single i.c.v. FGF1 injection (3 μg), both RMP and AP frequency of NPY/AgRP neurons from *Lep^ob/ob^* mice were significantly decreased compared with those from NPY/AgRP neurons of *Lep^ob/ob^* mice receiving i.c.v. saline [RMP, degrees of freedom {df}: *t*(43) = 2.021, *P =* 0.0495; AP frequency, *t*(43) = 2.091, *P =* 0.0424; Figure 3, G and H]. It is noteworthy that the AP frequency of NPY/AgRP neurons measured 2 weeks after i.c.v. FGF1 treatment in *Lep^ob/ob^* mice was comparable to that observed in i.c.v. saline-treated nondiabetic *Lep^ob/+^* mice. Stated differently, it appears that elevated baseline NPY/AgRP activity in these mice is normalized for at least 2 weeks following a single i.c.v. injection of FGF1.

These findings collectively demonstrate that the increased activity of NPY/AgRP neurons in the *Lep^ob/ob^* mouse model of T2D is reversed at least 2 weeks after i.c.v. injection of a single dose of FGF1 that reliably elicits sustained reversal of hyperglycemia in these animals (1, 2, 4). The remarkably long-lived duration of this inhibitory effect is compatible with a role for reduced NPY/AgRP neuron activity in the effect of FGF1 to induce sustained diabetes remission in *Lep^ob/ob^* mice, although additional studies are needed to test this hypothesis directly.

### FGF1 enhances GABAergic tone in NPY/AgRP neurons from Lep^ob/ob^ mice.

**Figure 4:**
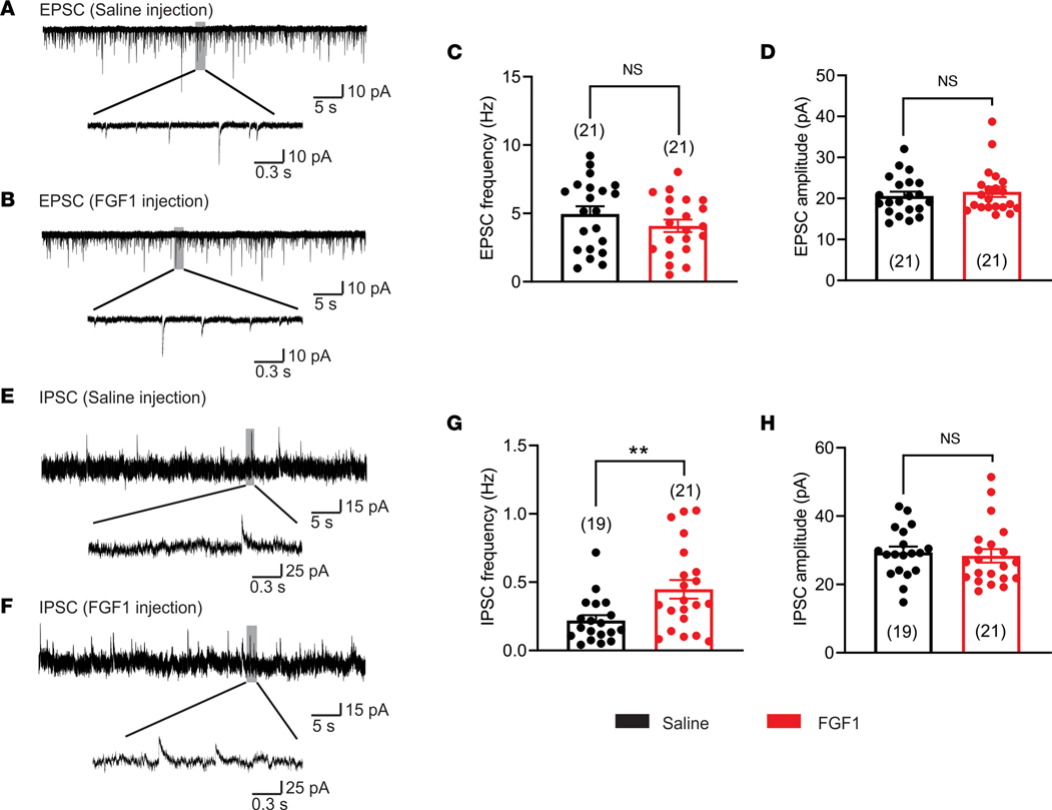
FGF1 increases an inhibitory input onto NPY/AgRP neurons in *Lep^ob/ob^* mice. (**A** and **B**) Voltage-clamp recording of excitatory postsynaptic currents (EPSCs) observed in arcuate NPY neurons from *Lep^ob/ob^* mice 2 weeks after i.c.v. injection of either (**A**) saline or (**B**) FGF1. (**C** and **D**) Histograms demonstrate the (**C**) average EPSC frequency and (**D**) amplitude of NPY neurons from male *Lep^ob/ob^* mice receiving i.c.v. saline (black, *n* = 21, from 3 mice) or FGF1 (red, *n* = 21, from 2 mice). (**E** and **F**) Voltage-clamp recording of inhibitory postsynaptic currents (IPSCs) observed in arcuate NPY neurons from *Lep^ob/ob^* mice after i.c.v. (**E**) saline or (**F**) FGF1 injection. (**G** and **H**) Histograms demonstrate the average (**G**) IPSC frequency and (**H**) amplitude of NPY neurons from male *Lep^ob/ob^* mice receiving i.c.v. saline (black, *n* = 19, from 3 mice) or FGF1 (red, *n* = 21, from 2 mice) injection. Data are taken from NPY neurons of male NPYhrGFP:*Lep^ob/ob^* mice and are expressed as mean ± SEM. **P < 0.01, unpaired *t* test compared with the saline group. The number of neurons studied for each group is in parentheses.

Based on our finding that the acute effect of FGF1 to inhibit ARC NPY/AgRP neurons requires GABAergic synaptic transmission (Figure 2C), we next asked whether a similar mechanism contributes to the sustained inhibition of these neurons in *Lep^ob/ob^* mice. To address this question, we monitored inhibitory and excitatory postsynaptic activity of NPY/AgRP neurons from NPY^hrGFP^:*Lep^ob/ob^* and *Lep^ob/+^* mice 2 weeks after injection of either saline or FGF1 (3 μg, i.c.v.). Similar to previous reports in juvenile mice (22), ARC NPY/AgRP neurons from *Lep^ob/ob^* mice receiving i.c.v. saline exhibited an increase in excitatory input (Figure 4, A–C) while also receiving less inhibitory input (Figure 4, E, F, and G) when compared with *Lep^ob/+^* mice (Supplemental Figure 5, C and G). These changes in synaptic activity were independent of changes in amplitude (Figure 4, D and H, and Supplemental Figure 5, D and H) and putatively contributed to the higher AP frequency in the former animals (Figure 3H and Supplemental Figure 4H).

While excitatory and inhibitory synaptic activity of NPY/AgRP neurons was not altered 2 weeks after i.c.v. FGF1 (3 μg) in *Lep^ob/+^* mice (Supplemental Figure 5), the frequency of inhibitory input to NPY/AgRP neurons from *Lep^ob/ob^* mice was increased following FGF1 administration [3 μg FGF1, i.c.v.; *t*(38) = 2.839, *P =* 0.0072, Figure 4G], while the amplitude remained unchanged [*t*(38) = 0.3869, *P =* 0.7010, Figure 4H]. The frequency and amplitude of excitatory synaptic activity to NPY/AgRP neurons from *Lep^ob/ob^* mice 2 weeks after i.c.v. FGF1 injection was also unaltered when compared with that of mice receiving saline [excitatory postsynaptic current (EPSC) frequency; *t*(40) = 1.213, *P =* 0.2322, EPSC amplitude; *t*(40) = 0.600, *P =* 0.5515, Figure 4, C and D). Together, these findings demonstrate that the long-lasting inhibitory effect of i.c.v. FGF1 administration on NPY/AgRP neuron activity is associated with (and putatively mediated by) increased GABAergic input onto these neurons, with no detectable change in excitatory input. This interpretation is also consistent with evidence that mRNA encoding FGF receptor 1 is expressed in only approximately 20% of NPY/AgRP neurons (123 of 644 total neurons, Supplemental Figure 6), implying a non-cell-autonomous mechanism underlying FGF1-induced inhibition of at least some of these neurons.

## Discussion

In this study, we report that in nondiabetic WT mice, the activity of NPY/AgRP neurons was rapidly reduced both following i.c.v. FGF1 injection in vivo (based on cFos expression in fasted mice) and after bath application of FGF1 in an ex vivo brain slice preparation (based on electrophysiological assessments). Perhaps more importantly, we also showed that in the *Lep^ob/ob^* mouse model of T2D, NPY/AgRP neuronal activity remained inhibited for at least 2 weeks following a single i.c.v. injection of FGF1 (relative to i.c.v. vehicle-injected controls) and, furthermore, that the underlying mechanism involved increased synaptic input from upstream GABAergic neurons. The unprecedented duration of this inhibitory effect makes it attractive as a potential mediator of sustained glucose lowering elicited by centrally administered FGF1, and future studies are warranted to test this hypothesis.

In normal mice, NPY/AgrRP neuron activity is subject to regulation by distinct nutrient-related signals that operate over multiple time scales (23–28). The most rapidly conveyed afferent input is simply the sight or smell of food, which in a fasted animal can inhibit NPY/AgRP neurons within seconds. Beyond this, afferent signals generated by the GI tract in response to nutrient ingestion act over an intermediate time scale (minutes to hours), whereas humoral signals generated in proportion to body fat mass and energy balance (such as insulin and leptin) provide a less dynamic and more continuous source of inhibitory tone (23, 26). The net effect of these various inhibitory inputs is that NPY/AgRP neurons tend to be relatively inactive unless/until the animal experiences a state of negative balance sufficient to threaten body fuel stores. In this setting, activation of NPY/AgRP neurons is both necessary and sufficient for the hyperphagic response that promotes positive energy balance and replenishes depleted fuel stores (29).

Under pathological conditions, however, activation of these neurons can cause both obesity and diabetes, and hyperactivity of these neurons is a common feature in rodent models of T2D (21). In diabetic *Lep^ob/ob^* mice, for example, NPY/AgRP neuron activity is increased compared with that in nondiabetic WT controls (21), a finding confirmed in this study. Moreover, the reduction of melanocortin signaling that results from excessive NPY/AgRP neuron activity in these animals is implicated in their obese, diabetic phenotype (30). Conversely, glucose homeostasis can be improved in diabetic animals by restoring intact melanocortin signaling (16, 31–33).

Based on these findings, increased melanocortin signaling is an attractive candidate mediator of the sustained antidiabetic action elicited by FGF1. Consistent with this notion, the MBH is both a crucial brain area for this FGF1 effect (3) and the principal area controlling melanocortin system tone. Moreover, hypothalamic levels of *Npy* and *Agrp* mRNA are reduced for up to 6 weeks following a single i.c.v. FGF1 injection in *Lep^ob/ob^* mice (16). Based on these considerations, we sought to determine if NPY/AgRP neurons are inhibited by FGF1 and, if so, whether the effect is sufficiently prolonged to qualify as a candidate mediator of FGF1’s sustained antidiabetic action. In support of this hypothesis, we report that these neurons are rapidly inhibited by FGF1 both in vivo and ex vivo and that in diabetic *Lep^ob/ob^* mice, this effect persists for at least 2 weeks following i.c.v. FGF1 injection (16). Highly durable inhibition of NPY/AgRP neurons likely contributes to the increased melanocortin signaling implicated in sustained diabetes remission induced by the central action of FGF1 (16).

In addition to their finding that bath application of FGF1 depolarizes POMC neurons in an ex vivo slice preparation (also predicted to increase melanocortin signaling), recent work from the Kievet laboratory indicates that FGF1 fails to consistently inhibit NPY/AgRP neurons (34). While the latter finding appears incongruent with the current results, we note that this assessment was based on analyses of these neurons as a population. When the response of individual NPY/AgRP neurons is considered, however, the number inhibited by FGF1 (34) is consistent with our own data. These findings suggest that NPY/AgRP neurons are not a monolithic population with respect to the response to FGF1 and raise the possibility that rather than acting on NPY/AgRP neurons themselves, the primary effect of FGF1 is on an upstream neuronal population that synapses onto NPY/AgRP neurons.

Consistent with this interpretation is our finding that FGF1-induced inhibition of NPY/AgRP neurons ex vivo is blocked by bath application of a GABAergic antagonist, implying that at least some effects of FGF1 on NPY/AgRP neurons are mediated indirectly. Further support for this hypothesis is provided by evidence that, whereas FGF receptors are highly expressed by glial cell types (35), *Fgfr1* mRNA is expressed by only a small fraction (~20%) of NPY/AgRP neurons. Moreover, blood glucose levels are minimally affected when FGFR1 is selectively deleted from NPY/AgRP neurons (36). While additional studies are necessary to identify upstream GABAergic neurons and establish their contribution to the inhibitory effect of FGF1 on NPY/AgRP neurons, we note that at the transcriptional level, glial cells — astrocytes and tanycytes in particular — are far more responsive to FGF1 than are neurons (16). Combined with evidence that cellular contacts between astrocytes and NPY/AgRP neurons increase markedly following i.c.v. FGF1 injection in *Lep^ob/ob^* mice (16), the possibility is raised that FGF1 action on glial cells contributes to the sustained inhibition of NPY/AgRP neurons that we observed. This hypothesis is also compatible with evidence of a role for PNNs in the response to FGF1, as most NPY/AgRP neurons are enmeshed by PNNs (18), and the abundance of these PNNs is reduced in the ARC of obese diabetic ZDF rats. Furthermore, not only does i.c.v. FGF1 injection restore these PNNs to normal, but intact PNNs appear to be required for sustained normalization of glycemia by i.c.v. FGF1 injection in these animals (19). Yet another piece of the FGF1 action puzzle involves the MAPK/ERK signal transduction pathway, which is required for proper neuron and glial function (37). This pathway is robustly activated in the MBH following i.c.v. FGF1 injection, and this activation lasts at least 24 hours (3). Furthermore, pharmacological blockade of this MAPK/ERK activation blocks sustained diabetes remission following i.c.v. FGF1 injection in *Lep^ob/ob^* mice (3). These findings collectively indicate that sustained remission of diabetic hyperglycemia following central FGF1 administration is dependent on sustained increases of melanocortin signaling, MAPK/ERK signal transduction, glial cell activation, and PNN reassembly in the MBH (3). Determining precisely where the highly durable inhibition of NPY/AgRP neurons fits into the sequence of events initiated by FGF1 is an important scientific priority.

The hypothesis that the inhibitory effect of FGF1 on NPY/AgRP neurons involves activation of GABAergic neurons that lie upstream is consistent with a large literature on the control of NPY/AgRP neuron activity (38–44). As one example, the rapid inhibition of NPY/AgRP neurons by food sensory cues is mediated by a subset of GABAergic neurons situated in the ventral aspect of the dorsomedial nucleus (DMN) that synapse onto NPY/AgRP neurons (38, 39, 42). While it is possible that these DMN neurons are direct targets for the action of FGF1, another possibility is that in T2D loss of PNN enmeshment has a destabilizing effect on synaptic input onto NPY/AgRP neurons, thus reducing tonic inhibition by GABAergic DMN neurons. In this scenario, the restorative effect of FGF1 on these PNNs could in theory help to reestablish inhibitory synaptic input onto NPY/AgRP neurons in a manner that can be sustained over time. Studies to test this hypothesis are underway.

In summary, we report that ARC NPY/AgRP neurons are inhibited following FGF1 administration both in vivo and ex vivo. This inhibitory effect is sustained for at least 2 weeks following a single i.c.v. FGF1 injection in the *Lep^ob/ob^* mouse model of T2D, and the underlying mechanism appears to involve increased inhibitory input from presynaptic GABAergic neurons. The highly durable nature of this inhibitory effect, combined with evidence linking NPY/AgRP neuron activation to the pathogenesis of hyperglycemia in diabetic animals, offers a potentially novel, feasible, and testable mechanism to explain sustained glucose-lowering elicited by i.c.v. FGF1 injection in murine models of T2D. Studies that test this hypothesis will be a priority moving forward.

## Methods

### Animals.

All animals were housed individually under specific pathogen–free conditions in a temperature-controlled environment (12-hour lights on/off cycle; lights on at 7:00 am) with ad libitum access to water and standard laboratory chow (LabDiet). C57BL/6J, NPY^hrGFP^, and *Lep^ob/ob^* (B6.Cg-Lep^ob^/J) mice were purchased from The Jackson Laboratory. Male *Agrp^Cre:GFP^* knockin mice (version 2) were generated by Richard Palmiter (University of Washington, Seattle, Washington, USA) (29). To identify NPY/AgRP neurons from *Lep^ob/ob^* and WT mice for electrophysiological recordings, NPY^hrGFP^ mice (20) were mated to *Lep^ob/ob^* (B6.Cg-Lep^ob^/J) mice to generate NPY^hrGFP^:*Lep^ob/ob^* mice (45).

### Cannulation surgeries.

Lateral ventricle cannulations (8IC315GAS5SC, 26 gauge, Plastics One) were performed under isoflurane anesthesia using the following stereotaxic coordinates: –0.7 mm posterior to bregma; 1.3 mm lateral, and 1.95 mm below the skull surface. Mice were treated perioperatively with Buprenorphine SR (Par Pharmaceutical) (1 mg/mL; 0.1 mL SQ per 25 g; 1 dose for 72 hours, Zoopharm LLC) and Carprofen (Zoetis Inc.) (1.3 mg/mL; 0.3 mL SQ per 25 g; 1 dose per 24 hours; 3 days; Zoetis Inc.) and then were allowed to recover for 2 weeks prior to being studied.

### i.c.v. injection.

Mean levels of blood glucose and body weight were matched between groups before i.c.v. injections. Animals received a single 3 μL i.c.v. injection of either murine FGF1 (1 μg/μL; a gift from Novo Nordisk) or 0.9% saline using a 33-gauge needle (Plastics One) extending 0.8 mm beyond the tip of the lateral ventricle cannula. For chronic studies, body weight and food intake measurements were taken every morning at 10 am (CST) for 3 days before injection and daily up to 2 weeks after injection.

### Immunofluorescence.

Dual-immunofluorescence histochemistry was performed to assess the effect of central administration of FGF1 on fasting-induced cFos induction in NPY/AgRP neurons. *Agrp^Cre:GFP^* mice underwent lateral ventricular cannulation followed by a 2-week period of recovery and habituation to daily handling. Habituated animals were then fasted for 24 hours and received a single i.c.v. injection of either vehicle or FGF1 (3 μg). Ninety minutes later, mice were anesthetized with ketamine and xylazine and perfused with ice-cold PBS followed by 4% paraformaldehyde in 0.1 M PBS, after which brains were removed. Anatomically matched free-floating coronal sections (30 μm thickness) from the rostral-to-caudal extent of the hypothalamus were collected, washed in PBS at room temperature, permeabilized in 0.1 % Triton X-100 and 0.1% BSA, and blocked in freshly prepared 5% normal serum (Jackson ImmunoResearch Laboratories). They were then incubated overnight at 4°C with rabbit anti-cFos antibody (1:100,000; PC38; Oncogene Research Products), followed by incubation in donkey anti-rabbit Alexa Fluor 555 (1:1,000; Molecular Probes Inc.). Sections were washed, reblocked in normal serum, and then incubated with chicken anti-GFP antibody (1:5,000; ab13970; Abcam), followed by incubation in goat anti-chicken Alexa Fluor 488 (1:1,000; Molecular Probes Inc.). Sections were then washed overnight in PBS and mounted Super-Frost Plus Slides (Fisher Scientific). Immunofluorescence images were captured using a Leica SP8X Scanning Confocal microscope with a HC FLUOTAR L ×25/0.95 W objective. Quantification of total ARC AgRP and cFos cell counts was performed using Qupath (https://qupath.github.io). Quantification of colocalization of cFos with Agrp neurons was performed by (a) exporting Qupath images to ImageJ (NIH) and converting them to binary images, (b) using an image calculator to multiply matched binary images and to identify AgRP neurons that contain cFos, and (c) quantifying of Agrp neurons that were cFos positive by using the analyze particles feature (46).

### Duplex ISH.

Mouse brains were prepared and stained as described previously (35). Briefly, mice were deeply anesthetized with ketamine and xylazine and were transcardially perfused with ice cold 4% PFA. Brains were extracted and postfixed overnight in 10% neutral buffered formalin. Tissues were subsequently paraffin processed, embedded in Surgiplast paraffin (Leica), sectioned at 5 μm intervals, and placed on to SuperFrost Plus glass slides (Fisher Scientific). Each slide included 2 sections of ARC separated by 750 μm, and we stained ARC pairs from 4 individual animals. Slides were stained on a Ventana Discovery ULTRA (Ventana Medical Systems) with RNAscope reagents (Advanced Cell Diagnostics), following technical bulletin no. 323300-USM-ULT. NPY mRNA was labeled with teal HRP using mouse-specific oligonucleotide probes (Mm-NPY, 313329, Advanced Cell Diagnostics), followed by staining of FGFR1 with fast red (Mm-FGFR1-O1-C2, 454949-C2, Advanced Cell Diagnostics) and a hematoxylin counterstain. Slides were coverslipped using EcoMount (Biocare) and imaged at ×20 on an AxioScan.Z1 (Zeiss AG). Post hoc analysis was performed in ImageJ (NIH) to quantify the number of dual-positive cells across samples. Our inclusion criteria for dual-positive cells included (a) visible nuclear staining with hematoxylin, (b) a minimum of 5 dots per perinuclear region of NPY, and (c) a minimum of 1 visible red dot to denote FGFR1 mRNA.

### Brain slice preparation.

To assess both the acute response of NPY/AgRP neurons to FGF1 ex vivo and activity of these neurons in *Lep*^ob/ob^ mice 2 weeks after i.c.v. FGF1 injection, brain slices were prepared from mice in which GFP is expressed under the control of the *Npy* promotor (NPY^hrGFP^ mice), as previously described (40, 41, 47–49). Briefly, male mice were deeply anesthetized with i.p. injection of 7% chloral hydrate and transcardially perfused with a modified ice-cold aCSF (described below) or high-sucrose aCSF (208 mM sucrose, 2 mM KCL, 26 mM NaHCO_3_, 10 mM glucose, 1.25 mM NaH_2_PO_4_, 2 mM MgSO_4_, 1 mM CaCl_2_ and 10 mM HEPES, PH = 7.3, ~310 mOsm). The mice were then decapitated, and the entire brain was removed and immediately submerged in ice-cold carbogen-saturated (95% O_2_ and 5% CO_2_) aCSF (124–126 mM NaCl, 2.8–5 mM KCl, 1.2 mM MgCl_2_ or 2 MgSO_4_, 1–2.5 mM CaCl_2_, 1.25–2.6 mM NaH_2_PO_4_, 0–26 mM NaHCO_3_, 0–10 HEPES, and 5–10 mM glucose). As the sagittal length of arcuate is approximately 1 mm, coronal sections were cut at 250 μm intervals to obtain 4 slices for recording from 1 mouse. Brain sections were made using a Leica VT1000S Vibratome and then incubated in oxygenated aCSF (32°C–34°C) for at least 1 hour before recording. The slices were bathed in oxygenated aCSF (32°C–34°C) at a flow rate of approximately 1.6 ml/minutes. All electrophysiology recordings were performed at room temperature.

### Whole-cell recordings.

The pipette solution for whole-cell recordings was as follows: 125 mM K-gluconate, 2–10 mM KCl, 10 mM HEPES, 5–10 mM EGTA, 1 mM CaCl_2_, 1 mM MgCl_2_, and 1–2 mM MgATP, 0–5 HEPES, 0.03 mM Alexa Fluor 350 hydrazide dye, pH = 7.3 for NPY^hrGFP^:*Lep^ob/ob^*. K-gluconate was replaced with equimolar Cs-gluconate for recording of spontaneous inhibitory postsynaptic currents (IPSCs) in response to acute FGF1 administration. Electrophysiological recordings were performed in a similar manner as in previous reports (40, 41, 50). Briefly, epifluorescence was used to target fluorescent cells, at which time the light source was switched to infrared differential interference contrast imaging to obtain the whole-cell recording (Zeiss Axioskop FS2 Plus equipped with a fixed stage and a QuanTEM:512SC electron-multiplying charge-coupled device camera). Electrophysiological signals were recorded using an Axopatch 700B amplifier (Molecular Devices), low-pass filtered at 2–5 kHz, and analyzed offline on a PC with patch-clamp (pCLAMP) electrophysiology data acquisition and analysis program (Molecular Devices). Membrane potentials and firing rates were determined from NPY neurons in brain slices. For acute drug administration, we targeted an NPY/AgRP neuron in 1 slice, and after recording switched to another slice to target the next NPY/AgRP neuron. Recording electrodes showed resistances of 2.5–5 MΩ when filled with the K-gluconate internal solution. Input resistance was assessed by measuring voltage deflection at the end of the response to a hyperpolarizing rectangular current pulse step (500 ms of −10 to −50 pA). Frequency and peak amplitude of excitatory and inhibitory currents were analyzed by using the Easy electrophysiology program (Easy Electrophysiology Ltd).

### Statistics.

For immunohistochemical experiments, data are shown as dot plots representing data from individual animals and bar graphs representing average ± SEM. Statistical analyses using unpaired 2-tailed Student’s *t* test was performed using R. For bath application studies, a change in membrane potential was required to be at least 2 mV in amplitude, and a change in activity was defined as a more than or equal to 25% change in action potential frequency that occurred in response to drug application. Membrane potential values were not compensated to account for junction potential (−8 mV). Effects of FGF1 on spontaneous IPSC frequency before and during acute FGF1 application were analyzed within a recording using the Kolmogorov-Smirnov test (a nonparametric, distribution-free goodness-of-fit test for probability distributions). All graphs and figures were generated using either GraphPad Prism 9.0 software or CorelDraw X8 (Corel Corp). All data from different groups were analyzed using an unpaired, paired, or multiple unpaired 2-tailed Student’s *t* test as well as 1-way ANOVA where appropriate. Results are reported as the mean ± SEM unless indicated otherwise, as indicated in each figure legend, where *n* represents the number of cells studied. Significance was set at *P* < 0.05 for all statistical measures.

### Study approval.

All experiments were performed in accordance with the guidelines established by the *Guide for the Care and Use of Laboratory Animals* (National Academies Press, 2011) and were approved by the University of Washington, Novo Nordisk Research Center Seattle, and the University of Texas Institutional Animal Care and Use Committees.

## Author contributions

Author order was determined using the following considerations. EH contributed most to this study and, therefore, is listed first. EH, JMS, AFB, AJM, THM, KLG, GJM, KWW, and MWS designed the experiments. EH, JMS, AFB, CMB, YD, DC, JMB, AJM, and BANP collected and analyzed the data. EH, JMS, AFB, AJM, KWW, and MWS wrote the manuscript. All authors reviewed and edited the manuscript. KWW and MWS are the guarantors of this work and, thus, had full access to all the data in the study and take responsibility for the integrity of the data and the accuracy of the data analysis.

## Supplementary Material

Supplemental data

## Version 1 08/02/2022

In-Press Preview

## Version 2 09/08/2022

Electronic publication
