# Supplementary material for: Sustained inhibition of NPY/AgRP neuronal activity by FGF1
Source: JCI Insight. 2022 Sep 8;7(17):e160891. doi: 10.1172/jci.insight.160891 (PMC9536267; doi:10.1172/jci.insight.160891)
Supplement: Supplemental data [file jciinsight-7-160891-s164.pdf]

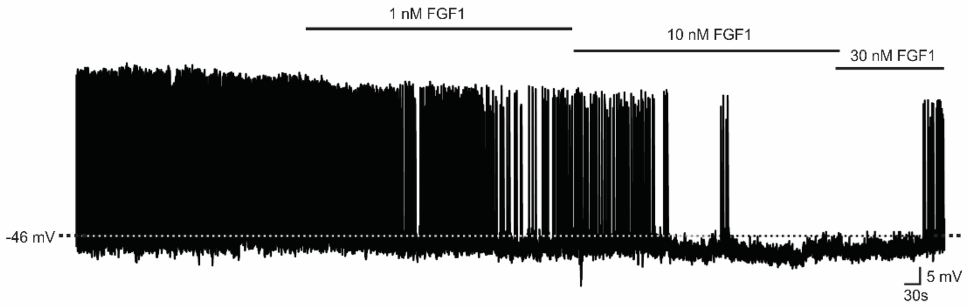

Supplementary figure 1 (related to Figure 2). FGF-1 reduced AP frequency of NPY/AgRP neurons in a concentration-dependent manner. Current clamp trace showing FGF-1 effects at 1, 10, and 30 nM in NPY/AgRP neurons (5 mice were used to generate data)**.**

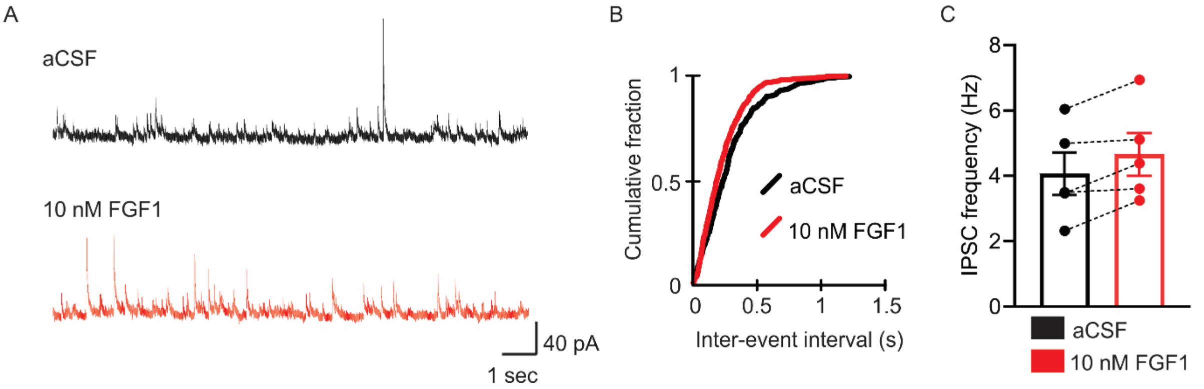

Supplementary figure 2 (related to Figure 2). Bath application of FGF1 enhances GABAergic tone onto NPY neurons in an ex vivo slice preparation. (A) Representative trace showing the spontaneous IPSC frequency of an NPY neuron before (black) and during (red) bath application of 10 nM FGF1. (B) Cumulative fraction plot shows a significant increase in sIPSC frequency in response to FGF1 (KS test; $P < 0.05$). (C) Histogram indicating FGF1-induced changes in sIPSC frequency observed in 5 neurons (from 4 slices in 1 mouse).

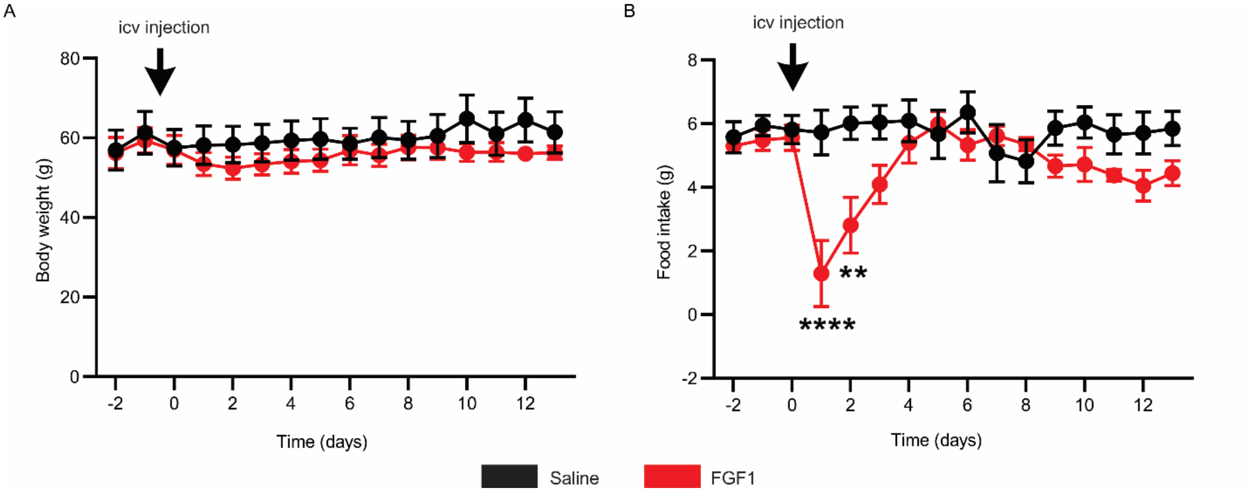

Supplementary figure 3 (related to Figures 3 and 4). A single injection of FGF1 (icv) transiently reduces food intake in *Lep*$^{ob/ob}$ mice. (A) Body weight curve and (B) food intake of male *Lep*$^{ob/ob}$ mice that received a single injection of FGF1 (red) or Saline (black) into the lateral ventricle. Data are from male *Lep*$^{ob/ob}$ mice (n=3-4) and are expressed as mean ± SEM. ** p<0.01 and **** p<0.0001, multiple unpaired t-test compared to Saline group.

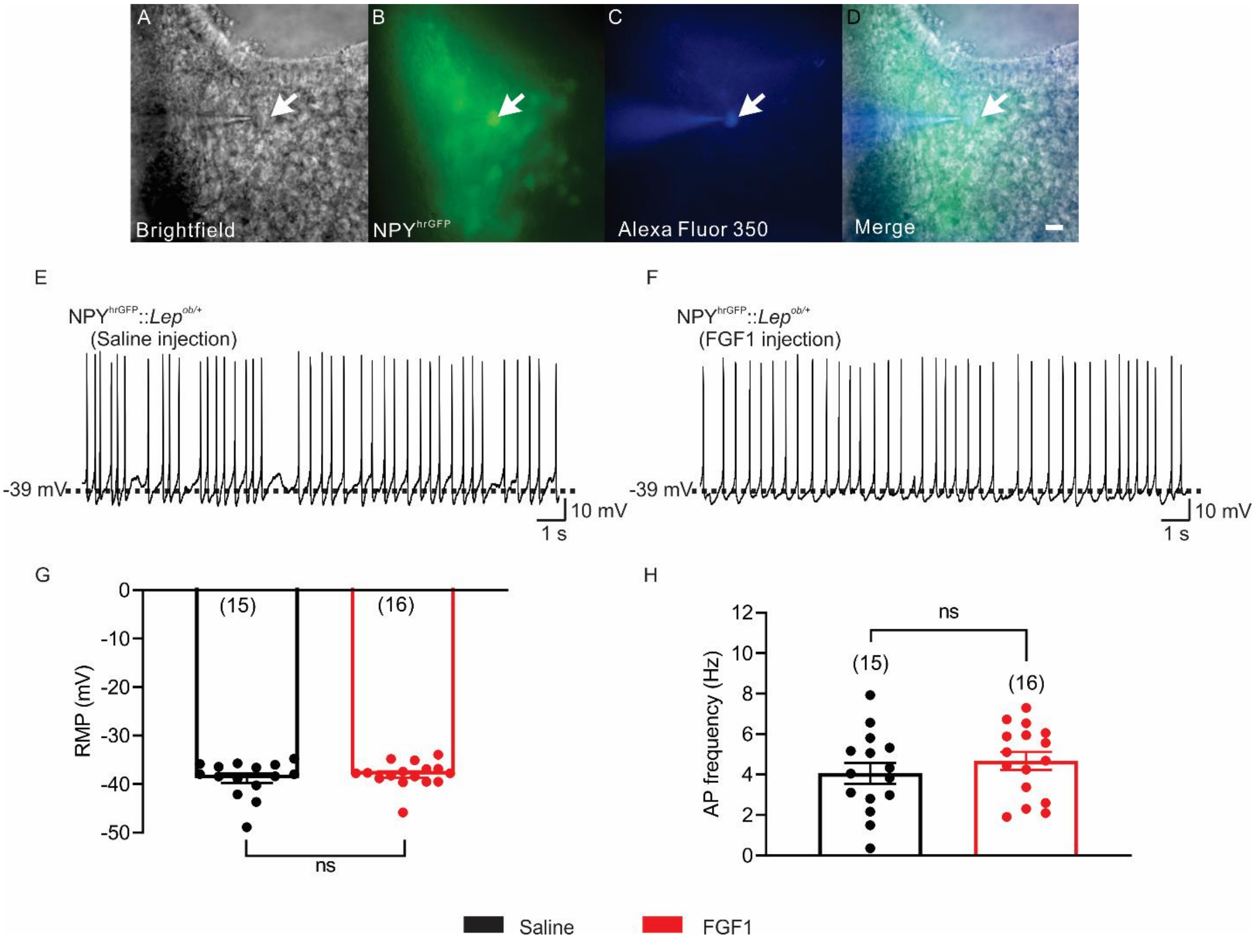

Supplementary figure 4 (related to Figure 3). NPY/AgRP neuronal activity from *Lep*$^{ob/+}$ mice remains unchanged 2 wk after icv injection of FGF1. Brightfield illumination (A) of NPY neurons from *Lep*$^{ob/+}$ mice. The same neuron under FITC (hrGFP; B) and Alexa Fluor 350 illumination (C). Merged image of targeted NPY neuron in shown in (D). Arrow indicates the targeted cell. Scale bar = 50 µm. Current-clamp recording shows the resting membrane potential of an NPY neuron from a male *Lep*$^{ob/+}$ mouse that received saline (E) or FGF1 (F). Histograms demonstrate the average resting membrane potential (G) and action potential frequency (H) of NPY neurons from male *Lep*$^{ob/+}$ mice following injection of saline (black; n=15, from 2 mice) or FGF1 (red; n=16, from 2 mice). Data are taken from NPY neurons of male NPY^{hrGFP}::*Lep*$^{ob/+}$ mice and are expressed as mean ± SEM. unpaired t-test compared to Saline group. The number of neurons studied for each group is in parentheses.

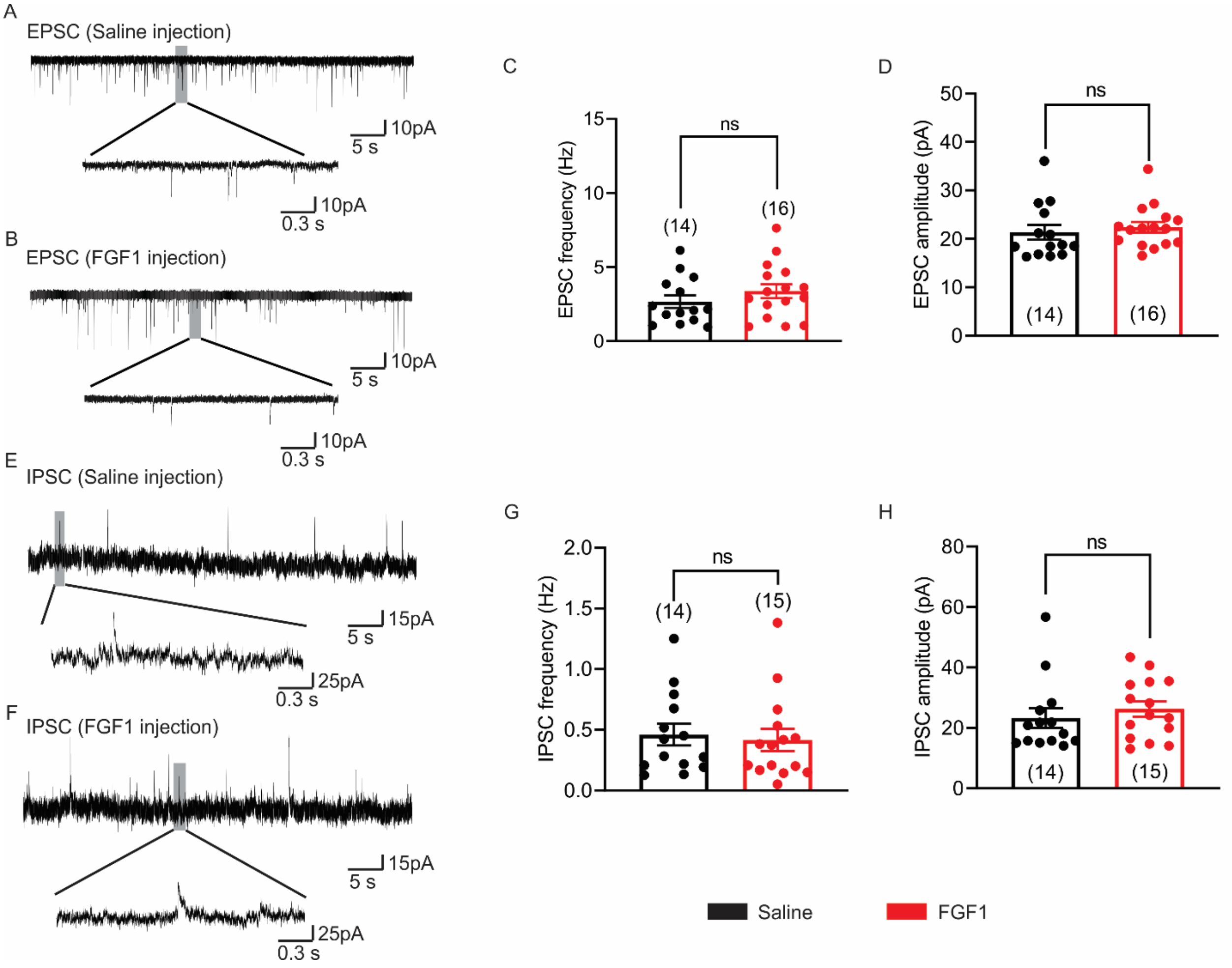

Supplementary figure 5 (related to Figure 4). Synaptic activity of NPY/AgRP neurons from *Lep*$^{ob/+}$ mice remains unchanged 2 wk after icv injection of FGF1. Voltage clamp recording of excitatory postsynaptic currents (EPSCs) observed in an NPY neurosn from *Lep*$^{ob/+}$ mice 2 wk after i.c.v. saline (A) or FGF1 (B) injection. Histograms demonstrate the average EPSC frequency (C) and amplitude (D) of NPY neurons from male *Lep*$^{ob/+}$ mice injected with either saline (black; n=14, from 2 mice) or FGF1 (red; n=16, from 2 mice). Voltage clamp recording of inhibitory postsynaptic currents (IPSCs) observed in an NPY neuron from *Lep*$^{ob/+}$ mice 2 wk after i.c.v. injection of saline (E) or FGF1 (F). Histograms demonstrate the average IPSC frequency (G) and amplitude (H) of NPY neurons from male *Lep*$^{ob/+}$ mice injected with saline (black; n=14, from 2 mice) or FGF1 (red; n=15, from 2 mice). Data are taken from NPY neurons

of male NPY^{hrGFP}::*Lep*$^{ob/+}$ mice and are expressed as mean ± SEM. unpaired t-test compared to Saline group. The number of neurons studied for each group is in parentheses.

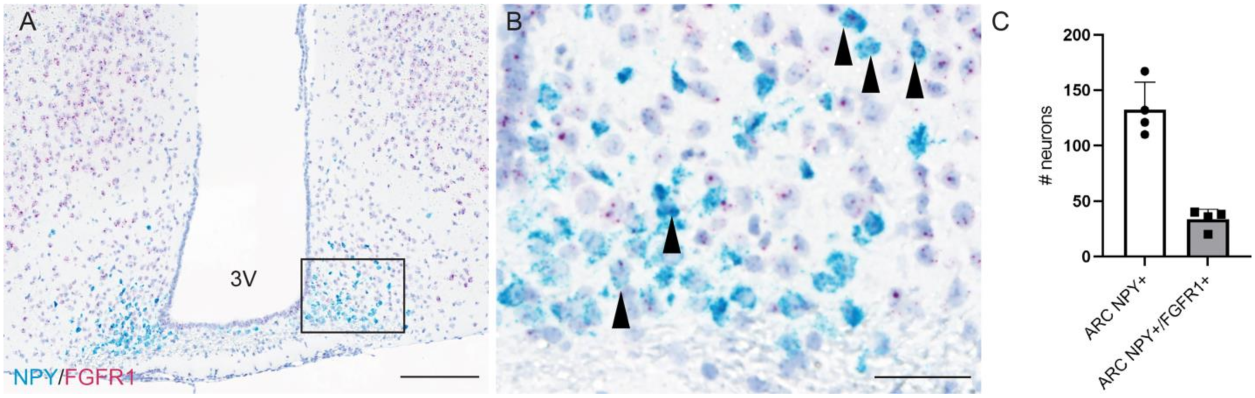

Supplementary figure 6. Co-expression of *FGFR1* mRNA in NPY neurons. A: Representative duplex ISH to *NPY* (teal) and *FGFR1* (red), with inset expanded in panel B. Arrows in panel B denote NPY neurons that contain *FGFR1* mRNA. C: <20% of NPY neurons (123/644, mean of 33.5 neurons per section) quantified in our analysis contain *FGFR1* mRNA whereas the majority of NPY neurons do not contain *FGFR1* mRNA (521/644 neurons, mean of 132.5 neurons per section). Data were analyzed from 2 hypothalamic sections from 2 separate mice. Scale bars in D = 200 µm, E = 50 µm.
